# Indirect Myocardial Injury in Polytrauma: Mechanistic Pathways and the Clinical Utility of Immunological Markers

**DOI:** 10.3390/jcdd12070268

**Published:** 2025-07-14

**Authors:** Makhabbat Bekbossynova, Timur Saliev, Murat Mukarov, Madina Sugralimova, Arman Batpen, Anar Kozhakhmetova, Aknur Zhanbolat

**Affiliations:** 1 Heart Center, Corporate Fund, University Medical Center, Nazarbayev University, Astana 010000, Kazakhstan; m.bekbosynova@umc.org.kz (M.B.); mmukarov@gmail.com (M.M.);; 2 Institute of Fundamental and Applied Medical Research, S.D. Asfendiyarov Kazakh National Medical University, Almaty 050000, Kazakhstan

**Keywords:** myocardial injury, inflammation, immunological markers, polytrauma, pathophysiology, diagnostics

## Abstract

Myocardial injury following polytrauma is a significant yet often underdiagnosed condition that contributes to acute cardiac dysfunction and long-term cardiovascular complications. This review examines the role of systemic inflammation, oxidative stress, neuro-hormonal activation, and immune dysregulation in trauma-induced myocardial damage. Key immunological markers, including interleukin-6 (IL-6), tumor necrosis factor-alpha (TNF-α), interleukin-1 beta (IL-1β), monocyte chemoattractant protein-1 (MCP-1), and adhesion molecules (ICAM-1, VCAM-1), are implicated in endothelial dysfunction, myocardial apoptosis, and ventricular remodeling. The interplay between these factors potentially exacerbates cardiac injury, increasing the risk of heart failure. Biomarker-guided approaches for early detection, combined with advanced imaging techniques such as speckle-tracking echocardiography and cardiac MRI, offer promising avenues for risk stratification and targeted interventions. Anti-inflammatory and oxidative stress-modulating therapies may mitigate myocardial damage and improve outcomes. This article highlights the clinical relevance of integrating immunological markers into diagnostic and therapeutic strategies to enhance the management of trauma-related cardiac dysfunction and reduce long-term morbidity.

## 1. Introduction

Myocardial injury following polytrauma is a significant yet often under-recognized complication that can lead to severe cardiovascular dysfunction and long-term morbidity [1]. While overt myocardial contusion or infarction is relatively rare and often clinically apparent, subclinical myocardial injury in polytrauma patients is frequently underdiagnosed due to the lack of specific cardiac monitoring. Several studies have reported elevated cardiac troponins and echocardiographic abnormalities in trauma patients without direct chest trauma, suggesting that systemic physiological stress may cause cardiac injury even when not clinically evident [2,3,4]. These findings underscore the potential significance of indirect myocardial injury, which may remain undetected but still contribute to adverse outcomes. 

While there is growing interest in understanding how systemic responses to trauma might affect cardiac function, the direct causal links between trauma-induced immune activation and myocardial injury remain incompletely understood. Some observational studies have shown elevated cardiac biomarkers in polytrauma patients, but distinguishing between direct myocardial involvement and nonspecific systemic effects remains a clinical challenge. Therefore, caution is needed in interpreting elevated immunological markers as definitive evidence of cardiac injury.

Polytrauma, defined as multiple severe injuries affecting different organ systems, triggers a complex physiological response that extends beyond direct mechanical damage [5]. Among the most affected organs in such cases is the heart, which becomes vulnerable to a cascade of pathological processes, including systemic inflammation, oxidative stress, neurohormonal activation, and immune dysregulation [6]. Unlike ischemic myocardial infarction, which primarily results from coronary artery occlusion, myocardial dysfunction in polytrauma is often mediated by a combination of hemodynamic instability, inflammatory cytokine release, mitochondrial dysfunction, and endothelial injury [7,8]. These mechanisms may contribute to acute myocardial impairment but also predispose patients to long-term complications such as heart failure and persistent cardiac remodeling.

Apart from direct injury, indirect mechanisms play a crucial role in cardiac dysfunction after polytrauma, including systemic inflammatory response syndrome (SIRS), oxidative stress, catecholamine surge. In cases of blunt chest trauma, cardiac contusion and microvascular dysfunction collectively exacerbate myocardial damage and impair recovery [9,10]. Among these, immune dysregulation and inflammation are increasingly recognized as central factors driving myocardial dysfunction in trauma patients.

Systemic inflammation following polytrauma is characterized by the massive release of pro-inflammatory cytokines, including interleukin-6 (IL-6), tumor necrosis factor-alpha (TNF-α), and interleukin-1 beta (IL-1β), which lead to endothelial activation, vascular permeability changes, and immune cell infiltration into the myocardium. This cytokine storm may contribute to a cascade of possible immune-mediated myocardial effects, contributing to myocardial apoptosis, fibrosis, and long-term ventricular dysfunction. Moreover, persistent low-grade inflammation in post-trauma patients has been linked to an increased risk of cardiac remodeling, heart failure, and arrhythmias, reinforcing the role of immunological factors in trauma-induced myocardial dysfunction.

Beyond cytokine-driven inflammation, oxidative stress plays a pivotal role in myocardial injury. Trauma-induced hypoxia, ischemia-reperfusion injury, and excessive neutrophil activation result in the overproduction of reactive oxygen species (ROS), leading to lipid peroxidation, mitochondrial dysfunction, and endothelial damage [11,12,13]. Elevated levels of myeloperoxidase (MPO), an oxidative stress biomarker, have been correlated with increased myocardial fibrosis and microvascular thrombosis in trauma patients [14,15]. The interplay between oxidative stress and immune activation may exacerbate cardiac stress and potentially contribute to injury, highlighting the need for early detection and intervention in high-risk trauma patients.

Another major contributor to myocardial dysfunction in polytrauma is neurohormonal activation, particularly the excessive release of catecholamines as part of the body’s stress response. This surge in epinephrine and norepinephrine leads to heightened myocardial oxygen demand, tachycardia, and increased vascular resistance, all of which can impair cardiac function [16]. Additionally, prolonged catecholamine exposure induces calcium overload in cardiomyocytes, triggering apoptotic pathways and further weakening cardiac contractility. In severe cases, this mechanism contributes to stress cardiomyopathy (Takotsubo cardiomyopathy), which has been observed in critically injured trauma patients.

Given the complex and multifactorial nature of myocardial injury in polytrauma, identifying key immunological and inflammatory biomarkers has become a priority in trauma research [17]. Specific markers such as IL-6, TNF-α, IL-1β, monocyte chemoattractant protein-1 (MCP-1), intercellular adhesion molecule-1 (ICAM-1), and vascular cell adhesion molecule-1 (VCAM-1) have been implicated in immune-mediated myocardial damage and may serve as valuable tools for early diagnosis, prognosis, and therapeutic targeting [17]. Elevated levels of these biomarkers correlate with increased myocardial stress, endothelial dysfunction, and long-term cardiovascular risk, suggesting that biomarker-guided approaches may improve trauma patient management and outcomes.

The article explores the role of immunological markers in myocardial injury following polytrauma, focusing on their pathophysiological significance, clinical relevance, and potential future applications. This review focuses predominantly on indirect mechanisms of myocardial injury in polytrauma patients. Direct myocardial injuries, such as cardiac contusions from blunt trauma, are outside the scope of this paper.

## 2. Pathophysiology of Myocardial Injury in Polytrauma

**Figure 1:**
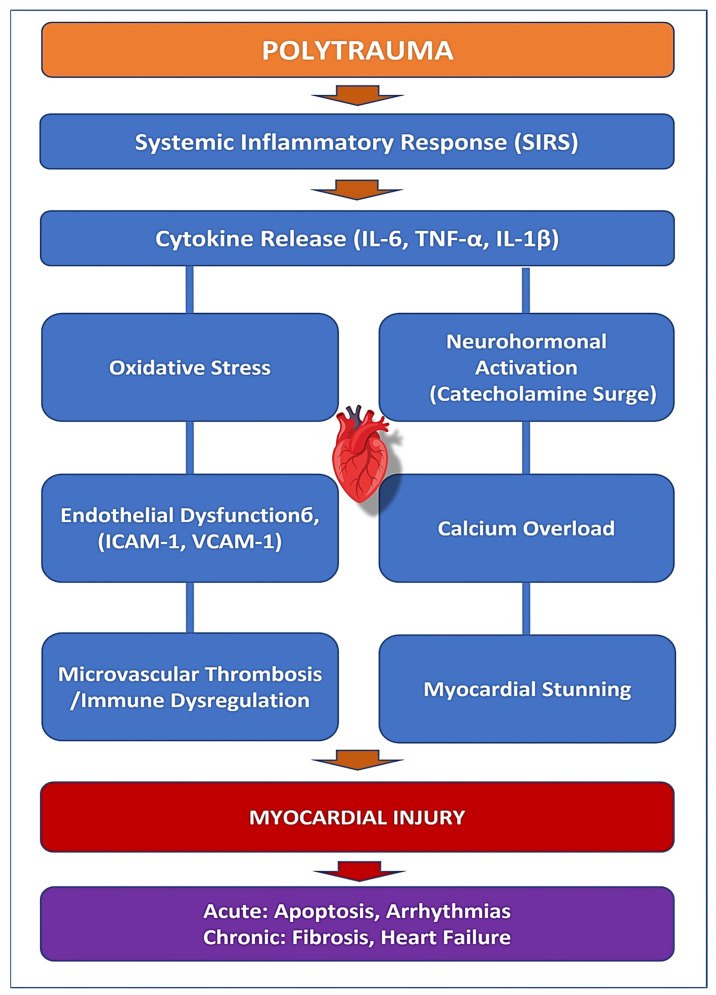
Pathophysiological cascade: from trauma to myocardial injury.

Myocardial injury following polytrauma is a complex and multifactorial process involving direct mechanical damage, systemic inflammation, oxidative stress, neurohormonal activation, and immune dysregulation [18,19] (Figure 1). Unlike ischemic myocardial infarction, which results primarily from coronary artery occlusion, myocardial dysfunction in trauma patients is often the result of hemodynamic, inflammatory, and metabolic disturbances that lead to acute and chronic cardiac impairment [20,21]. Understanding the intricate pathophysiology of post-traumatic myocardial injury is essential for early diagnosis, targeted intervention, and improved clinical outcomes.

Beyond direct trauma, systemic inflammation is hypothesized to contribute to myocardial stress following polytrauma. Although studies have demonstrated elevated cytokines such as TNF-α, IL-6, and IL-1β in trauma patients, the extent to which these contribute directly to myocardial dysfunction remains debated [22]. Some evidence suggests a correlation between inflammatory burden and elevations in cardiac biomarkers, but causality has not been conclusively demonstrated. For example, a prospective study by Ritter et al. (2024) evaluated cardiac biomarkers in polytrauma patients but found inconsistent associations with clinical evidence of myocardial dysfunction [17].

TNF-α promotes immune cell infiltration into the myocardium, impairing cardiac contractility and exacerbating oxidative stress [23]. IL-6 amplifies the acute inflammatory response and is closely associated with the severity of myocardial dysfunction. At the same time, IL-1β is a key driver of myocardial fibrosis and remodeling, increasing the risk of long-term cardiac complications [24,25]. The prolonged inflammatory state following polytrauma may worsen acute cardiac dysfunction and could increase the likelihood of chronic heart failure due to sustained myocardial stress (Figure 1).

Oxidative stress is another critical factor in the progression of myocardial injury after trauma (Figure 1). Hypoxia, ischemia-reperfusion injury, and excessive immune activation lead to an overproduction of reactive oxygen species (ROS), which cause damage to cardiomyocytes [26,27]. Mitochondrial dysfunction follows, impairing ATP production and triggering cell death pathways such as apoptosis and necrosis [28]. Myeloperoxidase (MPO), an oxidative stress marker released by activated neutrophils, is strongly linked to cardiac remodeling and long-term functional decline [29].

Neurohormonal activation, particularly the surge of catecholamines following trauma, further exacerbates myocardial stress (Figure 1). Excessive release of epinephrine and norepinephrine increases myocardial oxygen demand while simultaneously impairing oxygen supply. This sympathetic overdrive contributes to myocardial stunning, contractile dysfunction, and arrhythmias. Prolonged catecholamine exposure induces calcium overload in cardiomyocytes, triggering apoptosis and further weakening myocardial performance. This mechanism is particularly evident in stress-induced cardiomyopathies, such as Takotsubo cardiomyopathy, which have been observed in critically injured trauma patients.

Endothelial dysfunction plays a significant role in trauma-induced myocardial injury by promoting leukocyte adhesion, increasing vascular permeability, and contributing to microvascular thrombosis (Figure 1). The upregulation of adhesion molecules such as intercellular adhesion molecule-1 (ICAM-1) and vascular cell adhesion molecule-1 (VCAM-1) facilitates monocyte infiltration into the myocardium, triggering localized inflammation and worsening cardiac dysfunction [30,31]. The combination of endothelial dysfunction, oxidative stress, and hypercoagulability predisposes trauma patients to myocardial infarction, thrombosis, and organ failure, even in the absence of pre-existing coronary artery disease.

Immune dysregulation following polytrauma further complicates myocardial recovery (Figure 1). The initial hyperinflammatory state, driven by SIRS, is often followed by a compensatory anti-inflammatory response syndrome (CARS), characterized by immune suppression and increased susceptibility to infections [32,33]. This dysregulated immune response contributes to delayed myocardial healing, persistent inflammation, and fibrotic remodeling, increasing the risk of long-term cardiovascular complications. Persistent low-grade inflammation following trauma has been linked to progressive myocardial dysfunction and adverse cardiac outcomes, highlighting the need for targeted immunomodulatory interventions (Table 1).

The understanding of post-traumatic myocardial injury has significant clinical implications (Table 1). Identifying key immunological markers, such as IL-6, TNF-α, IL-1β, MPO, and adhesion molecules, could allow for early risk stratification and personalized therapeutic approaches [34,35,36]. Future research should focus on developing biomarker-guided strategies to predict myocardial dysfunction in trauma patients, enabling timely intervention. Anti-inflammatory therapies targeting IL-1β and TNF-α have shown promise in reducing cardiovascular complications and may be beneficial in mitigating trauma-related cardiac injury. Additionally, oxidative stress inhibitors, such as MPO-targeting agents, could be explored as potential therapeutic options to reduce cardiac remodeling and improve long-term outcomes.

Advancements in imaging techniques, including speckle-tracking echocardiography, could further enhance the early detection of myocardial dysfunction in trauma patients, allowing for pre-emptive management before the development of overt heart failure. Integrating biomarker profiling with advanced imaging modalities could provide a comprehensive assessment of myocardial injury, improving diagnostic accuracy and patient outcomes. Future studies should also explore the potential role of endothelial-stabilizing therapies and neurohormonal modulation in preventing post-traumatic cardiac complications.

While the precise timing and dynamics of inflammatory mediator release vary between individuals, a generalized sequence can be proposed based on current evidence. Within 0–6 h post-trauma, there is typically a sharp rise in IL-6, TNF-α, and IL-1β levels as part of the acute systemic inflammatory response. Between 6 and 24 h, elevation of endothelial adhesion molecules (ICAM-1, VCAM-1), increased vascular permeability, neutrophil infiltration, and rising myeloperoxidase (MPO) levels are observed. By 24–72 h, oxidative stress and mitochondrial dysfunction tend to peak, accompanied by the continued release of MCP-1 and sST2, along with the detection of cardiac biomarkers such as high-sensitivity troponin (hs-Tn). Beyond 72 h, secondary immune modulation (CARS), fibrotic signalling, and sustained low-grade inflammation may persist, contributing to adverse cardiac remodeling. This proposed timeline is intended to be illustrative and hypothesis-generating, and further studies are needed to validate cytokine and biomarker kinetics in trauma-specific cohorts.

Understanding the complex interplay between inflammation, oxidative stress, neurohormonal activation, and immune dysregulation in myocardial injury following polytrauma is essential for improving clinical outcomes. Targeting these pathophysiological pathways through early diagnosis, biomarker-based risk assessment, and novel therapeutic strategies has the potential to significantly enhance the management of trauma-related cardiac dysfunction, ultimately reducing mortality and improving quality of life in affected patients.

## 3. Key Immunological Markers in Myocardial Injury Following Polytrauma

The immune system plays a crucial role in myocardial injury following polytrauma, influencing both acute cardiac dysfunction and long-term remodeling. A complex interplay of pro-inflammatory cytokines, chemokines, adhesion molecules, and oxidative stress mediators contributes to endothelial dysfunction, immune cell infiltration, and myocardial damage. Identifying key immunological biomarkers associated with trauma-induced myocardial injury is essential for improving early diagnosis, risk stratification, and therapeutic interventions [35,36].

Among these markers, interleukin-6 (IL-6) has emerged as a central mediator of systemic inflammation following trauma. IL-6 levels rise rapidly after injury and correlate strongly with the severity of systemic inflammatory response syndrome (SIRS), myocardial dysfunction, and multi-organ failure [25,37]. By promoting endothelial activation and increasing vascular permeability, IL-6 facilitates immune cell infiltration into the myocardium, leading to oedema, oxidative stress, and eventual fibrosis. Its role in upregulating acute-phase proteins such as C-reactive protein (CRP) further links IL-6 to cardiovascular risk, making it a valuable biomarker for early assessment of myocardial stress in critically injured patients. Although IL-6 is a well-established marker of systemic inflammation, its specific role in mediating myocardial injury in polytrauma remains unclear. In many studies, elevated IL-6 is a general prognostic marker for disease severity rather than a specific indicator of cardiac damage.

Another key cytokine involved in myocardial dysfunction after trauma is tumor necrosis factor-alpha (TNF-α), a potent inflammatory mediator known to impair cardiac contractility and induce oxidative damage [38]. Elevated TNF-α levels have been associated with increased endothelial permeability, myocardial apoptosis, and mitochondrial dysfunction, all of which contribute to impaired myocardial function [24]. Additionally, TNF-α enhances the expression of adhesion molecules such as intercellular adhesion molecule-1 (ICAM-1) and vascular cell adhesion molecule-1 (VCAM-1), promoting leukocyte adhesion and infiltration into myocardial tissue, exacerbating local inflammation. Studies have shown that excessive TNF-α signalling can lead to the progression of myocardial fibrosis and heart failure, making it a potential therapeutic target in trauma-related cardiac dysfunction [24,25,38]. Interleukin-1 beta (IL-1β) further amplifies inflammatory signalling in myocardial injury by triggering cardiomyocyte hypertrophy, matrix remodeling, and fibrosis. IL-1β is a key driver of adverse cardiac remodeling, and its inhibition has been explored as a strategy to mitigate myocardial inflammation. The Canakinumab Anti-Inflammatory Thrombosis Outcomes Study (CANTOS) demonstrated that IL-1β blockade significantly reduced cardiovascular events, highlighting its relevance in post-traumatic myocardial injury [39].

Current support for IL–1β–targeted therapies in trauma-related myocardial injury is based on extrapolated evidence and should be interpreted accordingly. Moreover, it must be noted that most data on TNF-α/IL-1β/mediated myocardial injury come from sepsis, ischemia-reperfusion, or animal models. Direct evidence from polytrauma cohorts remains limited.

Beyond pro-inflammatory cytokines, cardiac-specific biomarkers provide insight into myocardial stress and damage in polytrauma patients. High-sensitivity troponin (hs-Tn), traditionally used to detect ischemic myocardial infarction, is now recognized as an important indicator of myocardial stress in trauma patients [40,41]. Unlike ischemic events, where troponin release is due to coronary artery occlusion, in trauma, hs-Tn elevation often reflects immune-mediated myocardial injury due to systemic inflammation and cytokine activation. Elevated hs-Tn levels correlate with IL-6 and IL-1β levels, indicating a strong link between inflammation and myocardial injury. Myeloperoxidase (MPO), an oxidative stress biomarker released by activated neutrophils, has been implicated in endothelial dysfunction, lipid peroxidation, and microvascular thrombosis [42]. MPO-mediated oxidative damage contributes to long-term cardiac remodeling, increasing the risk of heart failure [43]. The suppression of tumorigenicity 2 (sST2) protein has also emerged as a biomarker of myocardial strain and fibrosis, with elevated levels indicating poor cardiac recovery in trauma patients.

Chemokines such as monocyte chemoattractant protein-1 (MCP-1) further exacerbate myocardial inflammation by recruiting monocytes and macrophages to injured myocardial tissue [44]. MCP-1 is strongly linked to increased myocardial fibrosis and adverse cardiac remodeling, and elevated levels are associated with higher mortality in critically ill patients. Similarly, ICAM-1 and VCAM-1 facilitate leukocyte–endothelial interactions, promoting immune cell infiltration and worsening myocardial damage. These adhesion molecules are upregulated in response to TNF-α and IL-6, reinforcing the inflammatory cycle that drives myocardial dysfunction. Their persistent elevation is correlated with prolonged inflammatory activity and increased risk of heart failure [45].

Despite their usefulness in early diagnosis and risk stratification, many biomarkers lack specificity, are difficult to measure in routine clinical practice, or have limited availability. Future research should focus on refining biomarker-based strategies, integrating them with imaging technologies, and addressing accessibility issues to improve trauma-related cardiac care. Table 2 provides a comprehensive overview of key immunological biomarkers in myocardial injury following polytrauma, highlighting their roles, clinical significance, diagnostic potential, and limitations. 

The clinical relevance of these immunological markers lies in their potential for improving early diagnosis and guiding therapeutic strategies in post-traumatic myocardial injury [46]. Elevated levels of IL-6, TNF-α, IL-1β, hs-Tn, MPO, and MCP-1 can serve as early indicators of myocardial dysfunction, allowing for risk stratification and timely intervention. Integrating these biomarkers into clinical practice could facilitate the identification of high-risk trauma patients, enabling personalized treatment approaches [45]. Targeting inflammatory pathways through IL-1β and TNF-α inhibitors has shown promise in reducing cardiovascular complications, and further exploration of immunomodulatory therapies may provide additional benefits in trauma-related myocardial dysfunction. Antioxidant therapies aimed at reducing MPO-driven oxidative stress could also help mitigate cardiac remodeling and improve long-term outcomes.

Future research should focus on developing biomarker-based algorithms for early detection of trauma-induced myocardial dysfunction and integrating these markers with advanced imaging techniques such as speckle-tracking echocardiography to provide a more comprehensive assessment of myocardial injury. Identifying novel therapeutic targets within the inflammatory cascade could lead to improved treatment options that extend beyond traditional supportive care. The ability to monitor inflammatory biomarkers in real time may allow for the development of precision medicine approaches tailored to individual patients, ultimately improving survival rates and reducing the long-term cardiovascular burden of polytrauma. 

The growing understanding of immunological markers in myocardial injury following polytrauma underscores their critical role in early diagnosis, prognostication, and the development of novel therapeutic strategies, offering a promising avenue for improving outcomes in trauma patients. Table 2 includes biomarkers that are mechanistically plausible contributors to myocardial stress in polytrauma based on extrapolated data from inflammation and cardiovascular studies. However, not all markers have been validated specifically in the setting of trauma-induced cardiac dysfunction.

In addition to hs-Tn, heart-type fatty acid-binding protein (H-FABP) has shown promise as an early biomarker of myocardial injury [47]. H-FABP is a low-molecular-weight cytoplasmic protein rapidly released from cardiomyocytes within 1–3 h of injury, preceding troponin elevation [48]. Its short half-life makes it particularly useful in early-phase diagnostics, especially in trauma settings where ischemic and non-ischemic injuries overlap [49]. Recent evidence suggests that H-FABP may be more sensitive than troponin in detecting minor myocardial damage in polytrauma patients [17]. Other potential candidates include GDF-15 (Growth Differentiation Factor-15), a stress-induced cytokine linked to cardiac strain and inflammation [50,51]; uPAR (soluble urokinase plasminogen activator receptor), which reflects immune activation and tissue remodeling and has been associated with poor outcomes in trauma [52,53]; and sST2 (suppression of tumorigenicity 2), a marker of myocardial fibrosis and ventricular remodeling, useful in risk stratification [54]. These biomarkers, particularly when used in combination with hs-Tn and inflammatory cytokines, may improve the specificity and timing of myocardial injury detection in trauma patients

## 4. Clinical Relevance and Future Directions

While the potential clinical utility of immunological biomarkers in trauma-induced myocardial injury is promising, the evidence base is still emerging. Many of the proposed markers are derived from observational data, preclinical studies, or non-trauma cohorts, and further validation is required to confirm their diagnostic and prognostic utility in polytrauma populations.

**Figure 2:**
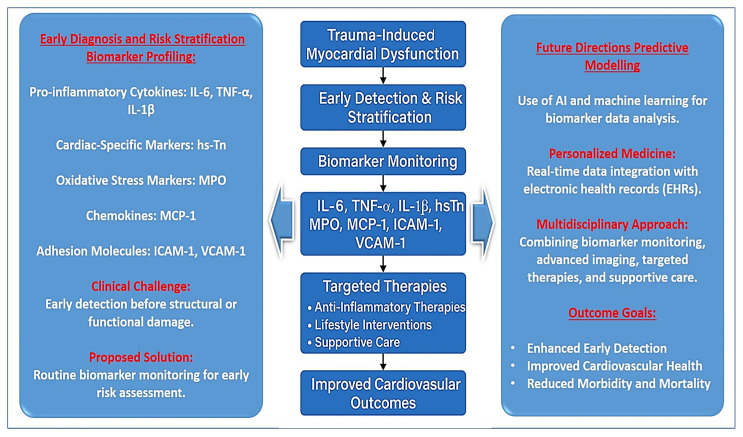
Clinical relevance and future directions in trauma-induced myocardial injury.

Trauma-induced myocardial dysfunction is often underdiagnosed due to its subtle and multifactorial presentation, yet it significantly impacts patient outcomes. The ability to measure specific inflammatory and oxidative stress markers, such as interleukin-6 (IL-6), tumor necrosis factor-alpha (TNF-α), interleukin-1 beta (IL-1β), high-sensitivity troponin (hs-Tn), myeloperoxidase (MPO), and monocyte chemoattractant protein-1 (MCP-1), can improve the accuracy of early detection and risk assessment in critically injured patients [45] (Figure 2).

One of the key clinical challenges in polytrauma management is the early identification of myocardial dysfunction, which often lacks clear clinical symptoms in the initial stages. Current diagnostic approaches, including electrocardiography (ECG) and echocardiography, may not always detect subtle myocardial injury. However, integrating biomarker profiling into clinical practice offers a more sensitive method for identifying myocardial stress and inflammation before significant structural or functional damage occurs. Elevated IL-6 and TNF-α levels within the first 24 h post-injury correlate strongly with worsening cardiac function and higher mortality rates, suggesting that routine monitoring of these markers could help identify high-risk patients early and allow for pre-emptive cardioprotective interventions. The use of cardiac troponin in conjunction with IL-6 and IL-1β levels could significantly improve the accuracy of myocardial injury detection in trauma patients, leading to timelier interventions and better long-term outcomes.

The integration of adhesion molecule biomarkers, such as intercellular adhesion molecule-1 (ICAM-1) and vascular cell adhesion molecule-1 (VCAM-1), into clinical risk stratification models also holds promise. These markers are early indicators of endothelial activation and leukocyte infiltration into the myocardium, processes that play a central role in trauma-induced myocardial inflammation. Studies suggest that persistently elevated ICAM-1 and VCAM-1 levels correlate with increased risk of cardiac fibrosis and ventricular dysfunction. Measuring these markers could help identify patients at risk of developing long-term cardiovascular complications, allowing for early implementation of anti-inflammatory and endothelial-stabilizing therapies (Figure 1).

From a therapeutic standpoint, the identification of these immunological markers opens up new avenues for targeted interventions. The use of anti-inflammatory therapies in trauma-related cardiac dysfunction is gaining attention, with several promising approaches under investigation. IL-1β inhibition, as demonstrated in the CANTOS trial, has shown significant reductions in cardiovascular events in high-risk patients, suggesting that IL-1β blockade could also be beneficial in trauma patients with persistent myocardial inflammation. Similarly, TNF-α inhibitors, which have been widely used in autoimmune diseases, may offer cardioprotective benefits in polytrauma patients by reducing endothelial dysfunction, leukocyte infiltration, and myocardial fibrosis. Future clinical trials should assess the efficacy of cytokine-targeted therapies in mitigating trauma-induced cardiac injury and preventing the progression to chronic heart failure.

Beyond pharmacological approaches, lifestyle and supportive interventions play an important role in improving long-term cardiac outcomes in trauma survivors. Rehabilitation programs that incorporate cardiovascular monitoring, exercise therapy, and inflammation-modulating nutrition may help reduce chronic inflammation and improve myocardial recovery [55,56]. Strategies such as the Mediterranean diet, rich in anti-inflammatory polyphenols and omega-3 fatty acids, have been shown to reduce systemic inflammation and oxidative stress, making them potentially beneficial adjuncts in trauma recovery [57]. Future research should explore whether dietary and lifestyle interventions could complement biomarker-guided therapies in improving long-term cardiovascular health in trauma patients.

Another important future direction is the use of advanced imaging techniques in conjunction with biomarker assessment to enhance diagnostic accuracy. Traditional echocardiography may not always detect early myocardial dysfunction, particularly in trauma patients with subclinical myocardial stress. However, speckle-tracking echocardiography (STE) and cardiac magnetic resonance imaging (MRI) offer more precise assessments of myocardial deformation, fibrosis, and microvascular dysfunction [58,59,60]. Integrating STE and MRI findings with biomarker profiling could provide a comprehensive evaluation of myocardial health, allowing clinicians to better predict long-term cardiac outcomes and tailor personalized treatment plans for trauma patients.

Despite increasing recognition of myocardial injury as a potential complication in polytrauma patients, the current evidence linking systemic inflammatory responses to direct cardiac dysfunction remains limited and primarily inferential. Many of the immunological markers discussed in this review, such as interleukin-6 (IL-6), tumor necrosis factor-alpha (TNF-α), interleukin-1 beta (IL-1β), and myeloperoxidase (MPO), have been extensively studied in the contexts of sepsis, cardiovascular disease, or general critical illness. However, there is a lack of robust, trauma-specific studies demonstrating that elevations in these markers directly cause myocardial injury in polytrauma populations. Most available data are observational and do not establish causality between immune activation and cardiac dysfunction. Furthermore, these markers may reflect overall physiological stress, tissue injury, or systemic inflammation, rather than myocardial involvement per se. As such, elevated cytokine levels should not be interpreted as definitive evidence of cardiac damage without supporting functional or imaging-based assessments.

Clinical validation of these markers in trauma-specific cohorts is essential. Future studies must focus on prospective, controlled designs that evaluate the relationship between biomarker trajectories and well-defined measures of cardiac function, such as echocardiographic changes, cardiac MRI findings, or validated composite outcomes. Additionally, multicenter trials assessing biomarker specificity, sensitivity, and prognostic value in diverse trauma populations are needed before widespread adoption in clinical practice.

Until such data are available, the clinical use of these markers should be approached with caution. They may serve as adjuncts to risk stratification and hypothesis generation but should not replace standard diagnostic modalities for detecting myocardial injury in trauma patients.

Furthermore, future research should focus on developing biomarker-based risk stratification models that incorporate machine learning and artificial intelligence (AI) to improve predictive accuracy and clinical decision-making [61,62] (Figure 2). AI-driven algorithms have the potential to analyze complex biomarker patterns, identify high-risk patients early, and recommend individualized treatment strategies based on real-time data [63,64]. The integration of AI into electronic health records (EHRs) could enhance early warning systems, ensuring that trauma patients with elevated inflammatory and oxidative stress markers receive timely cardioprotective interventions [64,65].

The growing understanding of immunological markers in myocardial injury following polytrauma underscores their critical role in clinical decision-making and therapeutic development. Routine monitoring of key biomarkers such as IL-6, TNF-α, IL-1β, hs-Tn, MPO, MCP-1, ICAM-1, and VCAM-1 could revolutionize the early detection and management of trauma-induced myocardial dysfunction, leading to personalized and targeted treatment approaches. As research advances, the integration of biomarker-based diagnostics, anti-inflammatory therapies, advanced imaging, and AI-driven predictive models will play a pivotal role in enhancing survival rates and improving the long-term cardiovascular health of trauma patients [66,67]. A multidisciplinary approach, combining biomarker monitoring, innovative therapeutics, and supportive care, represents the future of precision medicine in trauma-related cardiac dysfunction, ultimately reducing cardiovascular morbidity and mortality in this vulnerable patient population.

## 5. Perspectives and Limitations

The identification of key immunological and inflammatory markers—such as interleukin-6 (IL-6), tumor necrosis factor-alpha (TNF-α), interleukin-1 beta (IL-1β), monocyte chemoattractant protein-1 (MCP-1), myeloperoxidase (MPO), intercellular adhesion molecule-1 (ICAM-1), and vascular cell adhesion molecule-1 (VCAM-1)—has advanced our understanding of the mechanisms underlying cardiac dysfunction in trauma patients. These biomarkers may reflect the degree of systemic and myocardial inflammation, endothelial activation, and oxidative stress, all of which are implicated in adverse ventricular remodeling, myocardial fibrosis, and an increased risk of long-term heart failure.

Inflammation is believed to play a significant role in post-traumatic myocardial dysfunction. The excessive release of pro-inflammatory cytokines may contribute to sustained immune activation, potentially promoting myocardial stress, injury, and fibrotic remodeling. Elevated levels of IL-6 and TNF-α have been associated with trauma severity, impaired cardiac function, and increased mortality risk, suggesting their potential utility as early indicators of cardiac involvement in critically injured patients [68]. Similarly, IL-1β has been identified as a key driver of chronic inflammation and ventricular remodeling, further highlighting the importance of early intervention in trauma-induced cardiac injury [69].

Oxidative stress, another critical factor, exacerbates myocardial apoptosis, mitochondrial dysfunction, and endothelial damage. Biomarkers such as MPO provide additional prognostic value, as elevated levels indicate heightened oxidative stress, increased microvascular thrombosis, and long-term cardiovascular complications. Given the interplay between inflammation and oxidative stress, the development of targeted anti-inflammatory and antioxidant therapies represents a promising approach to reducing trauma-induced cardiac damage.

Despite growing awareness of immune-mediated myocardial dysfunction in trauma patients, early diagnosis remains a significant clinical challenge. Conventional cardiac assessments, such as electrocardiography (ECG) and echocardiography, may not adequately detect subtle immune-mediated myocardial injury. However, integrating biomarker profiling with advanced imaging techniques such as speckle-tracking echocardiography (STE) and cardiac MRI has the potential to enhance early detection and improve risk stratification [70,71]. Future studies should focus on refining these diagnostic approaches to allow for personalized treatment strategies based on biomarker-guided decision-making.

Beyond pharmacological interventions, lifestyle modifications and rehabilitation strategies should be considered essential components of post-traumatic cardiovascular care. Encouraging trauma survivors to adopt anti-inflammatory diets, engage in supervised exercise programs, and undergo regular cardiovascular monitoring may improve long-term heart health and prevent the progression to chronic heart failure. Future research should explore the combined impact of pharmacological, lifestyle, and rehabilitative interventions on myocardial recovery in polytrauma patients.

Looking ahead, the development of biomarker-based predictive models using artificial intelligence (AI) and machine learning algorithms holds promise for early risk stratification and personalized treatment approaches. AI-driven models could help analyze complex biomarker patterns, integrate imaging findings, and identify high-risk patients, allowing clinicians to tailor interventions more effectively [71]. The incorporation of biomarker-driven algorithms into electronic health records (EHRs) could enhance real-time clinical decision-making, ensuring that trauma patients at risk for myocardial dysfunction receive timely interventions.

While immunological and oxidative stress biomarkers hold significant promise for improving the early diagnosis and risk stratification of trauma-induced myocardial injury, several limitations currently impede their routine clinical implementation. One of the primary challenges is the limited specificity of these markers. Pro-inflammatory cytokines such as interleukin-6 (IL-6), tumor necrosis factor-alpha (TNF-α), and interleukin-1 beta (IL-1β) are elevated in a wide array of inflammatory and systemic conditions beyond cardiac injury, including infections, autoimmune disorders, and non-cardiac trauma-related inflammation. This lack of specificity complicates the interpretation of elevated biomarker levels and increases the risk of misattributing systemic inflammation to myocardial involvement in the absence of supporting clinical or imaging evidence.

In addition, most of the supporting data for these biomarkers are derived from studies in sepsis, ischemia-reperfusion models, or chronic cardiovascular disease. Direct evidence linking these markers to myocardial dysfunction in polytrauma patients remains limited. Without trauma-specific prospective studies that correlate biomarker levels with validated cardiac imaging findings or long-term outcomes, their diagnostic and prognostic utility in this context remains uncertain.

Another important barrier is the absence of standardized assays and reference ranges. Variability in laboratory techniques, reagent quality, and measurement platforms can lead to inconsistent results between institutions, undermining the reproducibility and clinical reliability of these tests. Furthermore, many of the assays used to quantify biomarkers such as myeloperoxidase (MPO), suppression of tumorigenicity 2 (sST2), or monocyte chemoattractant protein-1 (MCP-1) are technically demanding, costly, and not widely available outside of research or specialized centers. This limited availability poses particular challenges in emergency and critical care settings where rapid, accessible diagnostics are essential.

Operational constraints also limit the utility of these biomarkers in acute trauma care. Most are not suitable for point-of-care testing and require processing times that may not align with the urgent decision-making required in trauma resuscitation. The absence of established clinical workflows or guidelines for incorporating biomarker data into triage or therapeutic decision-making further complicates their implementation. Without clear algorithms or integration into electronic health record systems, the interpretation of these biomarkers remains largely dependent on individual clinician expertise and institutional practices.

Finally, the predictive power of these biomarkers as standalone tools is modest. Given the multifactorial nature of myocardial dysfunction in trauma, driven by hemodynamic instability, neurohormonal activation, immune dysregulation, and oxidative stress, biomarkers are most effective when used in conjunction with imaging modalities and comprehensive clinical assessment. Overreliance on biomarker levels in isolation may lead to oversimplification of complex pathophysiological processes and potentially suboptimal patient management.

## 6. Conclusions

There needs to be more awareness of the possibility of myocardial injury in polytrauma, as it is often underdiagnosed due to its subtle and multifactorial nature. Once myocardial injury is suspected or confirmed, clinicians must consider a broad range of possible etiologies. Importantly, trauma alone, even when accompanied by a systemic inflammatory response, does not necessarily indicate cardiac involvement. The mere presence of systemic inflammation should not be equated with myocardial damage without supporting clinical, biochemical, or imaging evidence. This review has highlighted the indirect pathways through which myocardial injury may occur following polytrauma, including immune dysregulation, oxidative stress, neurohormonal activation, and endothelial dysfunction. These possible mechanisms are complex and interconnected, but should not be assumed to be universally present in all trauma patients. Accurate diagnosis requires a combination of cardiac biomarkers (e.g., IL-6, TNF-α, hs-Tn), advanced imaging, and clinical judgment. The clinical implications are significant. Recognizing true myocardial involvement early can allow for timely, targeted interventions that may reduce long-term cardiac complications. However, the current lack of standardization in diagnostic criteria and variability in biomarker availability limit widespread application. Future research should focus on clarifying the specificity and predictive value of proposed biomarkers, validating imaging protocols, and distinguishing between systemic immune activation and direct cardiac injury. In particular, well-designed prospective trials are urgently needed to evaluate the temporal dynamics, diagnostic accuracy, and prognostic relevance of immunological and oxidative stress biomarkers in trauma-specific populations. These studies should aim to correlate biomarker trajectories with objective cardiac outcomes, such as imaging findings or clinical endpoints, to establish clinically meaningful thresholds and support evidence-based integration into trauma care pathways. Multicenter, longitudinal trials with standardized assay protocols and harmonized data collection will be essential for generating the high-quality evidence needed to guide clinical decision-making. Ultimately, a nuanced and evidence-based approach is needed to improve diagnosis and management. By integrating immunological insights with clinical vigilance and diagnostic precision, we can move toward more accurate risk stratification and personalized care for trauma patients with suspected cardiac involvement. Awareness, critical evaluation of possible causes, and avoidance of overinterpretation are key to translating these insights into meaningful clinical outcomes.

## Figures and Tables

**Table 1:** A summary of the main ideas and principles of the pathophysiology of myocardial injury following polytrauma.

Key Mechanism	Description	Clinical Implications	Early Detection & Diagnostics
Systemic Inflammation (SIRS)	Trauma triggers a massive inflammatory response with increased TNF-α, IL-6, and IL-1β, promoting endothelial dysfunction and myocardial apoptosis.	Increased risk of heart failure, prolonged inflammation, myocardial fibrosis, and remodeling.	Measurement of inflammatory biomarkers (IL-6, TNF-α, IL-1β), CRP levels, and echocardiography for early detection of myocardial dysfunction.
Oxidative Stress	Hypoxia, ischemia-reperfusion injury, and immune activation lead to excessive ROS production, mitochondrial dysfunction, and cell death.	Cardiac remodeling, endothelial dysfunction, increased long-term cardiovascular risk.	Detection of oxidative stress markers (MPO, ROS levels), lipid peroxidation assays, and mitochondrial function tests.
Neurohormonal Activation	Elevated catecholamines (epinephrine/norepinephrine) increase myocardial oxygen demand, causing contractile dysfunction, myocardial stunning, and arrhythmias.	Myocardial stunning, calcium overload, risk of Takotsubo cardiomyopathy in trauma patients.	Plasma catecholamine levels, HRV (heart rate variability) monitoring, echocardiography for myocardial function assessment.
Endothelial Dysfunction	Upregulation of adhesion molecules (ICAM-1, VCAM-1) increases leukocyte infiltration, leading to microvascular thrombosis and myocardial inflammation.	Increased risk of myocardial infarction, thrombosis, organ failure, even without coronary artery disease.	Endothelial function testing (flow-mediated dilation), measurement of ICAM-1 and VCAM-1 levels, and D-dimer tests for thrombotic risk.
Immune Dysregulation	Hyperinflammatory response (SIRS) is followed by compensatory immune suppression (CARS), delaying myocardial healing and increasing susceptibility to infections.	Persistent inflammation, fibrotic remodeling, risk of long-term cardiac complications.	Immune profiling (T-cell activation markers), cytokine level assays, and CRP monitoring for prolonged inflammation.
Biomarkers & Diagnostics	IL-6, TNF-α, IL-1β, MPO, and adhesion molecules can help in early risk stratification. Advanced imaging (e.g., speckle-tracking echocardiography) can enhance early detection.	Early intervention, personalized treatment strategies, improved diagnostic accuracy.	High-sensitivity cardiac troponin, echocardiographic strain imaging, and biomarker-based risk assessment tools.
Therapeutic Considerations	Anti-inflammatory therapies targeting IL-1β and TNF-α, oxidative stress inhibitors, endothelial stabilizers, and neurohormonal modulation are potential treatment options.	Reduction in cardiovascular complications, improved trauma-related cardiac outcomes, enhanced patient survival.	Serial biomarker tracking, cardiac imaging follow-ups, and therapeutic response monitoring using advanced analytics.

**Table 2:** The key immunological markers involved in myocardial injury following polytrauma.

Biomarker	Role in Myocardial Injury	Clinical Implications	Problems and Issues	Diagnosis and Treatment
Interleukin-6 (IL-6)	Central mediator of systemic inflammation, promotes endothelial activation, increases vascular permeability, and facilitates immune cell infiltration into the myocardium.	Correlates with severity of systemic inflammatory response syndrome (SIRS), myocardial dysfunction, and multi-organ failure.	Lacks specificity for myocardial injury; elevated in various inflammatory conditions.	Can serve as an early indicator of myocardial stress, guiding risk stratification and potential anti-inflammatory interventions.
Tumor Necrosis Factor-α (TNF-α)	Impairs cardiac contractility, increases endothelial permeability, induces apoptosis, and mitochondrial dysfunction. Upregulates ICAM-1 and VCAM-1, promoting leukocyte adhesion.	Contributes to myocardial fibrosis, endothelial dysfunction, and progression of heart failure.	Anti-TNF therapies have systemic effects and may suppress immune responses, increasing infection risk.	Targeting TNF-α with inhibitors may help reduce inflammation-driven myocardial damage.
Interleukin-1 Beta (IL-1β)	Triggers cardiomyocyte hypertrophy, matrix remodeling, and fibrosis. Amplifies inflammatory signalling in myocardial injury.	Linked to prolonged hospital stays and increased mortality in trauma patients.	IL-1β inhibitors are costly and may not be widely available for trauma patients.	IL-1β inhibitors (e.g., Canakinumab) have shown promise in reducing cardiovascular complications.
High-Sensitivity Troponin (hs-Tn)	Biomarker of myocardial stress; elevated in trauma patients due to immune-mediated myocardial injury rather than coronary occlusion.	Strong correlation with IL-6 and IL-1β levels; serves as a predictor of myocardial dysfunction.	May be elevated due to non-cardiac causes, including renal dysfunction and muscle damage.	Useful in differentiating trauma-induced cardiac stress from ischemic myocardial infarction.
Myeloperoxidase (MPO)	Released by activated neutrophils, contributes to endothelial dysfunction, oxidative stress, and microvascular thrombosis.	Increases risk of long-term cardiac remodeling and heart failure.	Difficult to measure in routine clinical practice; oxidative stress is influenced by multiple factors.	MPO-targeting therapies may help mitigate oxidative damage and improve myocardial recovery.
Suppression of Tumorigenicity 2 (sST2)	An indicator of myocardial strain and fibrosis, associated with poor cardiac recovery.	Predicts adverse cardiac outcomes in trauma patients.	Not yet widely adopted in clinical settings; limited availability of standardized testing.	Potential biomarker for early identification of high-risk trauma patients.
Monocyte Chemoattractant Protein-1 (MCP-1)	Recruits monocytes and macrophages to injured myocardial tissue, exacerbating inflammation and fibrosis.	Linked to increased mortality in critically ill patients.	High variability in MCP-1 expression; influenced by multiple inflammatory pathways.	Could be a therapeutic target for reducing myocardial inflammation and fibrosis.
Intercellular Adhesion Molecule-1 (ICAM-1)	Facilitates leukocyte adhesion and infiltration into the myocardium, worsening local inflammation.	Associated with persistent inflammation and prolonged myocardial dysfunction.	Lacks specificity; elevated in various conditions, including infections and autoimmune diseases.	Monitoring ICAM-1 levels may help assess the severity of inflammatory myocardial injury.
Vascular Cell Adhesion Molecule-1 (VCAM-1)	Upregulated in response to TNF-α and IL-6, promoting leukocyte recruitment and endothelial dysfunction.	Contributes to chronic myocardial inflammation and heart failure progression.	Measurement of VCAM-1 levels is not routine; requires specialized laboratory testing.	May serve as a biomarker for assessing vascular inflammation in trauma patients.

## Data Availability

No new data were created or analyzed in this study. Data sharing is not applicable to this article.
